# Age-Related Cognitive Decline, Focus on Microbiome: A Systematic Review and Meta-Analysis

**DOI:** 10.3390/ijms241813680

**Published:** 2023-09-05

**Authors:** Donatella Coradduzza, Stefania Sedda, Sara Cruciani, Maria Rosaria De Miglio, Carlo Ventura, Alessandra Nivoli, Margherita Maioli

**Affiliations:** 1 Department of Biomedical Sciences, University of Sassari, 07100 Sassari, Italy; donatella.coradduzza0@gmail.com (D.C.); stefania.sedda8@gmail.com (S.S.); scruciani@uniss.it (S.C.); 2 Department of Medicine, Surgery and Pharmacy, University of Sassari, 07100 Sassari, Italy; demiglio@uniss.it (M.R.D.M.); anivoli@uniss.it (A.N.); 3 Laboratory of Molecular Biology and Stem Cell Engineering, National Institute of Biostructures and Biosystems-Eldor Lab, Innovation Accelerator, CNR, Via Piero Gobetti 101, 40129 Bologna, Italy; 4 Center for Developmental Biology and Reprogramming (CEDEBIOR), Department of Biomedical Sciences, University of Sassari, Viale San Pietro 43/B, 07100 Sassari, Italy

**Keywords:** aging, biomarkers, cognitive decline, gut microbiome, neurodegenerative disorders, microbial diversity, relative abundance, systematic review, meta-analysis

## Abstract

Aging is a complex process influenced by genetics and the environment, leading to physiological decline and increased susceptibility to diseases. Cognitive decline is a prominent feature of aging, with implications for different neurodegenerative disorders. The gut microbiome has gained attention for its potential impact on health and disease, including cognitive function. This systematic review and meta-analysis aimed to investigate the relationship between the gut microbiome and cognitive function in the context of aging. Following PRISMA guidelines, a comprehensive search strategy was employed in PubMed, Scopus, and Web of Science databases. Studies exploring the role of the microbiome in cognition and neurodegenerative disorders, published between 2013 and 2023, were included. Data extraction and quality assessment were performed. Quantitative synthesis using statistical analyses was performed to examine microbial diversity and relative abundance in various cognitive conditions. Sixteen studies involving a total of 1303 participants were included in the analysis. The gut microbiota’s relative abundance was different in individuals with cognitive impairments such as Alzheimer’s disease, Parkinson’s disease, and dementia, compared to the healthy controls. The most prevalent phyla affected were Firmicutes, Bacteroidetes, Actinobacteria, and Proteobacteria. Meta-analyses indicated substantial heterogeneity among studies focusing on Alzheimer’s disease. The overall quality of evidence related to microbial analysis was moderate. The gut microbiome’s role in cognitive decline and neurodegenerative disorders warrants investigation. Altered microbial abundance, particularly in specific phyla, is associated with cognitive impairments. However, variations in study findings and methodologies highlight the complexity of the relationship between the gut microbiome and cognitive function. Further studies are needed to better understand the mechanisms underlying this connection and its potential implications for aging and cognitive health.

## 1. Introduction

Aging, an intrinsic feature of all living beings, is the process related to the time-dependent deterioration of the physiological functions necessary for survival and fertility [1,2]. It is a complex process influenced by both genetic and environmental factors [3,4]. The rate of aging can vary among individuals, so the biological age may not always align with the chronological age [5].

The impact of demographic aging, a transition towards a much older population structure within Western countries, and higher life expectancy are transforming the shape of the age pyramid [6,7,8]. Data shows that over the decade from 2012 to 2022, the share of the population aged 65 years or over increased by more than one-fifth (21.1%). This means that, in the Western population, over 21.1% of people were aged 65 and over [9].

Changes associated with aging include sensory changes, muscle weakening, reduced mobility, fat changes, and increased susceptibility to diseases such as hypertension, cardiovascular diseases, diabetes, osteoarthritis, osteoporosis, cancer [10,11,12], neurological disorders, and cognitive decline [13,14]. The relationship between aging and cognitive decline is, today, well established.

This decline can include changes in memory, executive function, processing speed, and reasoning.

Cognition and an individual’s resulting cognitive function encompass numerous processes, notably life-long learning [15]. This spans quantitative reasoning and memory function, alongside several other long- and short-term processes [16]. The current literature describes that, originally, cognition was hypothesized to be solely regulated by the central nervous system (CNS), with damage to the CNS causing cognitive decline [17,18]. However, more recently, it has become clear that cognition is impacted by several organ systems and processes, including the immune system and the gut microbiome [19]. Parkinson’s disease and Alzheimer’s disease represent two of the most common neurodegenerative disorders, with global prevalence rates of more than 10 million and 32 million, respectively [20,21]. The gut microbiome has been indicated to play a role in the pathogenesis of both disorders.

The gut microbiome has been a prime focus of research over the past decade due to its important role in human health and disease, with evidence demonstrating its influence in the development of chronic diseases [22].

The impact of microbiome bacteria and their metabolites on disease states constitutes a multifaceted and continually evolving realm of investigation. Although not all disease mechanisms have been fully elucidated, certain overarching concepts can be delineated, including the relationship between dysbiosis and immune dysregulation. Dysbiosis refers to an imbalance in the composition of the microbiome, characterized by an overrepresentation of specific microorganisms alongside a decline in others. This imbalance can precipitate immune dysregulation, wherein the immune system’s capability to differentiate between harmful pathogens and beneficial microorganisms becomes disrupted. This dysregulation can contribute to the hallmark chronic inflammation observed in numerous diseases, including autoimmune conditions.

Another significant mechanism involves molecular mimicry, whereby certain microorganisms or their components share structural resemblances with host tissues. Consequently, the immune system erroneously targets its own tissues due to the presence of analogous molecules on pathogens. This mechanism is believed to play a pivotal role in autoimmune diseases.

Additionally, microorganisms within the gut can produce a diverse array of metabolites through their metabolic processes. These metabolites can enter the bloodstream, affecting tissues in distant locations. Some metabolites exhibit anti-inflammatory properties, while others promote inflammation. An illustrative example is the production of short-chain fatty acids (SCFAs) by specific gut bacteria, known for their anti-inflammatory attributes and their influence on immune cell function.

The integrity of the gut barrier, constituted by cells lining the intestines, plays a pivotal role in averting the passage of detrimental substances from the gut into the bloodstream. Dysbiosis can compromise this barrier’s robustness, facilitating the entry of toxins, pathogens, and microbial metabolites into the circulation. This influx can stimulate immune responses and contribute to systemic inflammation.

Furthermore, components of the microbiome have the capability to directly activate immune cells via pattern recognition receptors. These receptors discern distinct molecular patterns on microorganisms, thereby initiating immune responses. Excessive or prolonged activation of these responses can culminate in chronic inflammation and tissue damage.

Crucially, the regulation of T regulatory cells is not to be overlooked. Certain gut bacteria are believed to foster the development and activity of regulatory T cells (Tregs), pivotal in restraining immune responses and maintaining immune tolerance. Dysbiosis has the potential to disrupt this equilibrium, potentially fueling autoimmune diseases. It is noteworthy that these mechanisms can display variations contingent on the specific disease and the microorganisms implicated.

The microbiome encompasses an array of entities, covering bacteria, archaea, viruses, and eukaryotes [23]. This diverse community has also emerged as a pertinent factor in cognitive decline, with recent evidence suggesting that the gut microbiota might serve as a susceptibility factor for Alzheimer’s disease [24].

In the realm of age-related cognitive decline, several bacterial phyla and their associated families and genera have emerged as key players in shaping gut-brain interactions (Table 1). The dynamic interplay between these microbial communities and cognitive health has prompted extensive investigation.

Firmicutes, a diverse group of bacteria, constitute a significant bacterial phylum within the human gut microbiome. Their roles span critical functions in gut health and metabolism. Notably, their adeptness in breaking down complex carbohydrates and producing short-chain fatty acids (SCFAs) has garnered attention. SCFAs, with their ability to bolster gut barrier integrity and induce anti-inflammatory responses, are instrumental contributors to overall well-being.

SCFAs, particularly those generated by Firmicutes, are believed to exert influence over the gut-brain axis, potentially impacting cognitive function. SCFAs possess the capability to modulate the immune system and confer neuroprotective attributes. Disruptions in SCFA production, attributed to shifts in Firmicutes populations, may compromise these advantageous impacts, potentially contributing to cognitive decline.

The potential decrease of beneficial Firmicutes bacteria could conceivably promote systemic inflammation and hinder effective gut-brain communication pathways. This intricate interplay underscores the need to comprehend the roles of Firmicutes and their associated metabolites in shaping cognitive health, while highlighting their potential as avenues for therapeutic interventions.

Among them, *Faecalibacterium prausnitzii*, a notable bacterium within the Firmicutes phylum of the human gut microbiome, is recognized for its role in maintaining gut health and overall well-being. This bacterium produces SCFAs, including butyrate, during dietary fiber fermentation. Their potential impact on brain health, particularly in neurodegenerative conditions such as Alzheimer’s disease, has attracted considerable interest.

SCFAs, notably butyrate, are believed to possess anti-inflammatory and neuroprotective properties that could potentially influence cognitive function. They may impact pathways related to oxidative stress, inflammation, and neurotrophic factors, all of which are essential for cognitive health and its potential decline.

Furthermore, Lactobacilli, a beneficial group within Firmicutes, have been subjects of interest in cognitive health and decline research. Lactobacilli’s ability to produce various metabolites, including SCFAs and neurotransmitter precursors, suggests potential implications for brain health. Similarly, Bacteroidetes’, another major bacterial phylum, capacity to break down complex carbohydrates and modulate immune responses aligns with their potential role in age-related cognitive decline.

Actinobacteria, although less understood in the context of cognitive decline, have been associated with the breakdown of complex carbohydrates and the production of bioactive compounds. Changes in Actinobacteria abundance and diversity have been observed in individuals with cognitive decline, including Alzheimer’s disease. Actinobacteria-derived metabolites, particularly those related to carbohydrate metabolism, might contribute to the gut-brain axis and cognitive function. These metabolites can impact inflammation and signaling pathways relevant to cognitive health.

Understanding the roles of bacterial phyla such as Firmicutes, Bacteroidetes, Actinobacteria, and Proteobacteria, along with their associated families and genera, sheds light on their potential influence on age-related cognitive decline.

Similarly, alterations to the prevalence of short-chain fatty acid-producing bacteria have been identified in patients with Parkinson’s disease and cognitive impairment [25]. Moreover, the intestinal microbiome is able to affect normal aging, frailty, and cognition decline.

The gut microbiome composition is associated with mortality in older individuals. A low uniqueness index and high representation of *Bacteroides* are independently linked to mortality, while the centenarian microbiome is characterized by a high abundance of *Lactobacilli* and *Bifidobacteria*.

Frailty, sarcopenia, and cognitive decline are associated with reduced gut microbiota biodiversity, reduced abundance of bacteria that synthesize short-chain fatty acids (SCFA), such as *Faecalibacterium Prausnitzii*, and reduced levels of fecal butyrate [22]. Older adults exhibit reduced pathways related to carbohydrate metabolism and amino acid synthesis, while oldest-old adults exhibit functional differences that distinguish their microbiota from that of young-old adults, as a greater potential for short-chain fatty acid production and increased butyrate derivatives [4,23,24,25].

The gut microbiome composition may contribute to healthy aging and longevity. Longevity may be characterized by increased flexibility and stability of the gut microbiota, as well as a balance between pro- and anti-inflammatory activity [4,26].

The gut microbiome changes associated with healthy aging may start in mid-life (around 40–50 years old) and are associated with a clear blood metabolomic signature. These changes may directly contribute to health as we age, by reducing inflammation in the gut [27,28,29,30]. No less interesting is how dietary intervention, particularly the Mediterranean diet, and exercise training are associated with improved biodiversity of the gut microbiota, increased capability of SCFA synthesis, and a related potential protection against frailty and cognitive decline [31].

Alterations in gut microbiota contribute to cognitive decline in aging through different mechanisms, including inflammation, oxidative stress, and the modulation of neurotransmitters and neurotrophins [32].

The composition and diversity of the gut microbiota may reflect different aging trajectories, and interventions such as dietary changes and exercise training can potentially improve gut microbiota health, mitigating age-related conditions. The gut microbiota plays a critical intrinsic role in the communication between an individual’s gut and brain, enabling it to influence the brain and trigger the production of neurotransmitters and neurotrophins [33]. As a result, the gut microbiome can modulate several inflammatory processes within the human body amongst other important mechanisms [34,35,36,37]. As depicted by Komanduri et al., “this close mutualistic relationship between the bacteria and the host indicates an important role of bacteria in biological and psychological features of the host” [38]. The role of the gut microbiota on cognition is, therefore, unsurprising, with evidence suggesting that gut microbiota interventions improve cognition and brain function, with these improvements observed in visuospatial memory, verbal learning and memory, and elements of attentional vigilance [39].

Despite the growing interest in the human microbiome and its role in pathogenesis and disease, the relationship between gut microbiota and cognitive function remains a controversial topic [40]. In a study performed in the U.S., microbiome diversity was lower in dementia patients as compared to healthy controls [41]. Nevertheless, in a Japanese population, an opposite situation was observed [42]. The present systematic review and meta-analysis aim to further investigate and dissect the relationship between the cognition function and microbiome within the context of aging.

## 2. Methods

This systematic review was performed in accordance with the Preferred Reporting Items for Systematic Reviews and Meta-Analyses (PRISMA) guidelines and was compliant with the Grades of Recommendation, Assessment, Development, and Evaluation (GRADE) criteria [43,44].

### 2.1. Search Strategy

A comprehensive search strategy was devised and employed for the literature databases PubMed, Scopus, and Web of Science between 1 May and 8 May 2023. The keywords “microbiome”, “gut microbiota”, “cognition”, “Alzheimer’s disease”, “Parkinson’s disease”, “dementia”, and “neurodegenerative disorder” were used to refine the scope of the literature identified in the initial searches.

### 2.2. Inclusion and Exclusion Criteria

The literature was then evaluated for relevance and was included if it explored and reported on the role of the microbiome and gut microbiota on cognition. This includes its role in the pathogenesis of neurodegenerative disorders, such as Parkinson’s disease and Alzheimer’s disease. Eligibility restrictions were also applied, with literature being excluded if it did not include human participants, were review articles, were published prior to 2013, or were published in any non-English language. The reference lists of identified literature and previous review articles were also screened to obtain additional relevant material.

### 2.3. Study Selection and Data Extraction

All of the identified literature was imported into EndNote X9 for ease during the screening process. Titles and abstracts were initially screened for eligibility by an independent author. Full-text records were further assessed using the inclusion and exclusion criteria by the same independent author. Manual searches of the reference lists of the identified records were also performed. Reported data in tabular and graphical form were extracted, cleaned, and tabulated from the full-text reports. The corresponding authors of the included studies were contacted to request any missing data. The literature that met the eligibility criteria and was included in this review was subject to data extraction by the independent author. The primary data points included study details (author and date), the study design, the study participants, the biospecimen collected, microbiological analyses, and the overall findings.

### 2.4. Outcome Measures

The primary outcomes of interest were measures of microbial diversity, namely changes in α-diversity and relative abundance of various taxa. For the purpose of this review, microbiome diversity refers to the variety and abundance of a given bacterial species in a defined unit of study [45,46]. In this study, the relative abundances at the phylum, family, and genus levels were examined. If required and necessary, units were converted so that related outcomes were on consistent scales.

### 2.5. Statistical Analysis

Data were extracted in Microsoft Excel and exported to STATA version 14.0 software where all statistical analyses were performed. For quantitative synthesis, continuous outcomes were reported using the standard mean difference. Three or more studies measuring the same outcome were combined with an inverse-variance weighted random-effects model. Forest plots were generated for the primary outcome of interest, divided by individual phyla, and with the weight indicating the influence of an individual study on the pooled result. Heterogeneity was quantified using τ2 (tau2) and I2 statistics.

### 2.6. Risk of Bias and Quality Assessment

The quality of the included literature was evaluated in concordance with GRADE criteria for determining the quality of evidence and recommendations for use [47]. These criteria consider the methodological quality, directness of evidence, heterogeneity, precision of effect estimates, and the risk of publication bias. This yielded a score of a high, moderate, or low level of evidence and recommendation for use.

## 3. Results

### 3.1. Identification of the Literature

**Figure 1:**
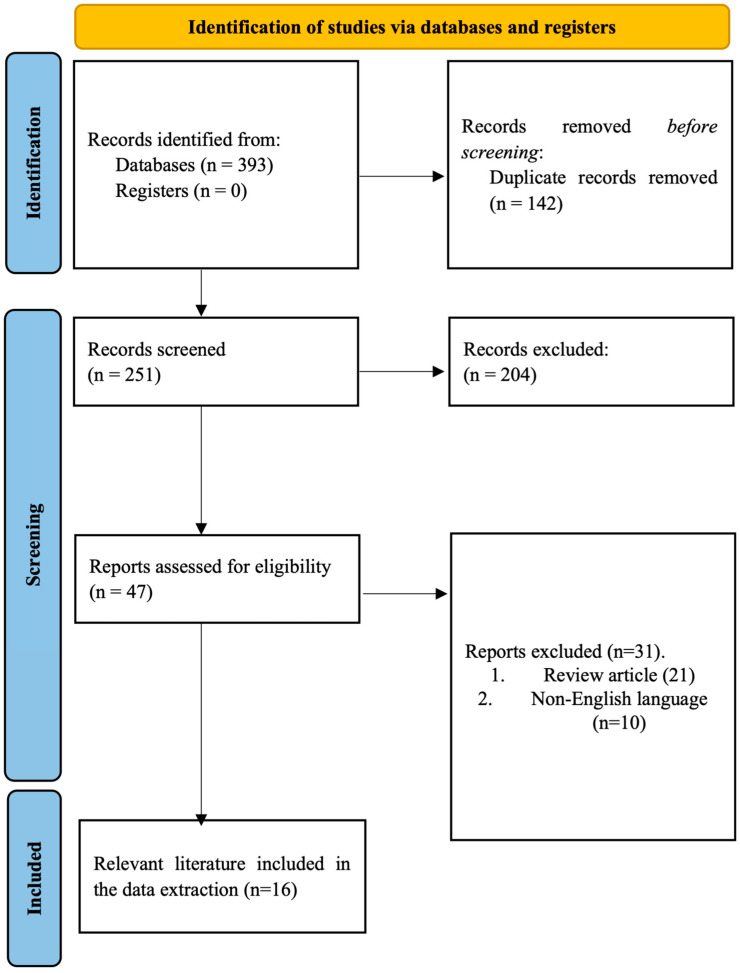
PRISMA flow diagram depicting the literature search and study selection process.

Implementation of the search strategy described 393 records, which were initially identified and deemed relevant to the research question. Following the removal of duplicate records in EndNote X9, the titles and abstracts of 251 records were screened against the inclusion and exclusion criteria. At this stage, a total of 204 records were excluded. The remaining 47 records were assessed as full-text articles for eligibility, with 31 being removed as they were either review articles or published in a non-English language. Consequently, 16 full-text articles were found to be relevant and included in the qualitative and quantitative analysis of this systematic review and meta-analysis (Figure 1).

### 3.2. Study Characteristics

All of the included literature investigated the relationship between cognitive impairment and gut microbiota. A total of 1303 patients were included and comprised 651 patients with Alzheimer’s disease or dementia, 232 patients with Parkinson’s disease, 33 patients with brain amyloidosis, 30 with post-stroke cognitive impairments, and 357 healthy or matched controls. The methodology followed a similar pattern across the included literature. The biospecimen collected in all studies was fecal samples and the most prevalent microbiological analyses performed were 16S rRNA sequencing (nine studies, 56%), T-RFLP analysis (two studies, 13%) and qRT-PCR (two studies, 13%).

### 3.3. Relative Abundance

**Figure 2:**
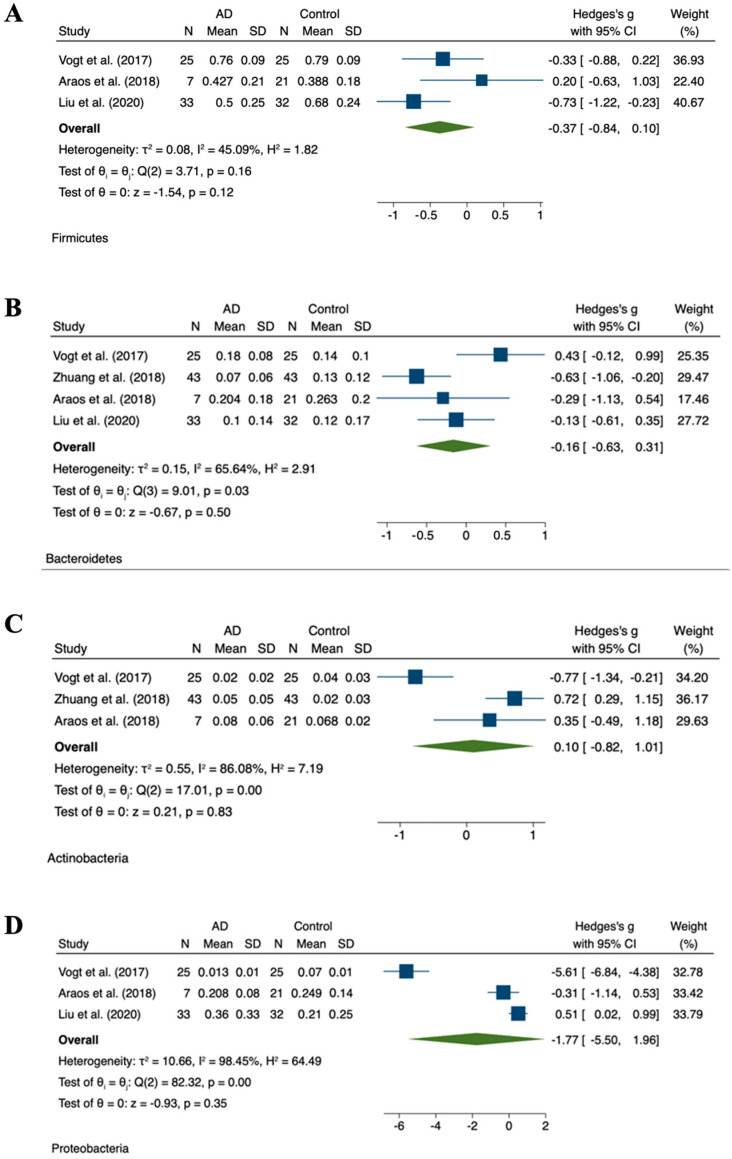
Meta-analysis of reported relative abundances in AD cohorts. Forest plots for relative abundance of phyla (**A**) *Firmicutes* [41,48,49], (**B**) *Bacteroidete* [41,48,49,50], (**C**) *Actinobacteria* [41,48,50], (**D**) *Proteobacteria* [41,48,49].

The abundance of fecal microbiota was altered between individuals with cognitive impairments and healthy controls. This was evident in individuals with Parkinson’s disease, Alzheimer’s disease, brain amyloidosis, and dementia, in addition to individuals with mild cognitive decline (Table 2). Differences in four phyla were primarily observed in the current literature between individuals with neurodegenerative conditions and healthy controls. Namely *Firmicutes*, *Bacteroidetes*, *Actinobacteria*, and *Proteobacteria*. The most dominant phyla in individuals with Alzheimer’s disease or dementia were *Bacteroidetes* and *Firmicutes.* Four studies reported on the relative abundance of the four main phyla in the microbiome of individuals with Alzheimer’s disease as compared to healthy controls [2,19,20,21]. However, no studies reported on the relative abundance of these phyla in individuals with Parkinson’s disease, brain amyloidosis, or mild cognitive decline. In individuals with Alzheimer’s disease, the meta-analyses were marked by considerable heterogeneity (Figure 2).

**Table 2:** Data extraction and summary of the human studies investigating the relationships between gut microbiota dysbiosis and cognitive impairment.

Author	Study Design	Study Subjects	Biospecimen	Microbiological Analysis	Findings
Cattaneo et al. [51]		*n* = 73 cognitively impaired patients (*n* = 40 with and *n* = 33 without brain amyloidosis), *n* = 10 HC	Fecal samples		Cognitively impaired patients with brain amyloidosis showed lower abundance of *Eubacterium rectale* and higher abundance of *Eschierichia/Shigella* as compared to both HCs and cognitively impaired patients without brain amyloidosis.
Minato et al. [52]	Prospective cohort	*n* = 36 PD (*n* = 18 deteriorated and *n* = 18 stable groups based on the degree of worsening of total UPDRS scores in 2 years)	Fecal samples	qRT-PCR of bacterial 16S or 23S rRNA	Low *Bifidobacterium* and *Bacteroides fragilis* at year 0 were associated with worsening of UPDRS scores in 2 years. Low *Bifidobacterium* at year 0 was associated with worsening of hallucinations/delusions in 2 years. Low *B*. *fragilis* at year 0 was associated with worsening of motivation/initiative in 2 years. The deteriorated group had lower *Bifidobacterium*, *B*. *fragilis*, and *Clostridium leptium* than the stable group at year 0.
Vogt et al. [41]		*n* = 25 AD, *n* = 25 HC	Fecal samples	16SrRNA sequencing	A decreased level of *Firmicutes* and *Bifidobacteria* and an increased level of *Bacteroidetes* in the fecal microbiota of dementia patients as compared to the controls.
Araos et al. [48]	Retrospective analysis	*n* = 362 advanced dementia	Fecal samples	16SrRNA sequencing	Among this patient population, the overall microbiome diversity was substantially lower than reported values of among HCs.
Nguyen et al. [53]		*n* = 4 AD	Fecal samples	16SrRNA genes sequencing	A remarkable variety amongst the small group of butyrate-producing bacteria was observed in the gut of elderly Japanese patients diagnosed with AD.
Saji et al. [54]	Cross-sectional	*n* = 34 dementia, *n* = 94 without dementia	Fecal samples	T-RFLP analysis	The number of *Bacteroides* (enterotype I) was lower and the number of “other” bacteria (enterotype III) was higher in dementia as compared to non-dementia patients. Multivariable analyses showed that the populations of enterotype I and enterotype III bacteria were strongly associated with dementia, independent of the traditional dementia biomarkers.
Qian et al. [55]		*n* = 45 PD, *n* = 45 HC	Fecal samples	High-throughput iluminia miseq sequencing targeting the V3-V4 region of 16S rRNA gene	Abundance of *Bifidobacterium*, *Butyricicoccus*, and *Clostridium* XIVb in gut microbiota was negatively correlated with the presence of cognitive impairment in patients with PD.
Zhuang et al. [50]		*n* = 43 AD, *n* = 43 HC	Fecal samples	16S rRNA sequencing	Several bacteria taxa in AD patients were different from those in the controls at taxonomic levels, such as *Bacteroides*, *Actinobacteria*, *Ruminococcus*, *Lachnospiraceae*, and *Selenomonadales*.
Haran et al. [56]	Prospective cohort	*n* = 51 no dementia *n* = 24 AD *n* = 33 other dementia types	Fecal samples	Shotgun metagenomics and mixed modeling rather than 16S rRNA	Increased proportions of *Bacteroides* spp., *Alistipes* spp., *Odoribacter* spp., and *Barnesiella* spp. and decreased proportions of *Lachnoclostridium* spp. were present in AD elders, while increased proportions of *Odoribacter* spp. and *Barnesiella* spp. and decreased proportions of *Eubacterium* spp., *Roseburia* spp., *Lachnoclostridium* spp., and *Collinsella* spp. were seen in elders with other dementia types.
Liu et al. [49]	Prospective and cross-sectional	*n* = 33 AD *n* = 32 pre-onset stage aMCI. *n* = 32 HC	Fecal samples	16S rRNA miseq sequencing	Proportion of phylum firmicutes was significantly reduced, whereas Proteobacteria was highly enriched in the AD as compared to HC. Similar alterations were observed at the order, class, and family levels of these two phyla. *Gammaproteobacteria*, *Enterobacteriales*, and *Enterobacteriaceae* showed a progressive enriched prevalence from HC to aMCI and AD patients.
Saji et al. [57]	Cross-sectional	*n* = 61 MCI without dementia *n* = 21 NC function	Fecal samples	T-RFLP analysis	Patients with MCI had a higher prevalence of *Bacteroides* and more likely to present with white matter hyperintensity and high voxel- based specific regional analysis system for AD. A multivariable logistic regression analysis revealed that a greater prevalence of *Bacteroides* was independently associated with MCI.
Ren et al. [58]	Cross-sectional	*n* = 13 PD with MCI (PD-MCI) *n* = 14 PD with NC (PD-NC) *n* = 13 HC	Fecal samples	16S rRNA sequencing and gas chromatography-mass spectrometry	Compared with HC and patients with PD-NC, the gut microbiota of patients with PD-MCI was significantly altered, particularly manifesting in enriched genera from the *Porphyromonadaceae* family and decreased the abundance of the genera *Blautia* and *Ruminococcus*.
Liu et al. [59]	Longitudinal	*n* = 30 PSCI *n* = 35 non-PSCI	Fecal samples	16SrRNA gene sequencing and gas chromatography- mass spectrometry	PSCI patients had disturbed microbial composition, and corresponding metabolites compared with non-PSCI patients. Increased *Fusobacterium* and deficiency of microbial metabolized SCFAs were significantly associated with PSCI.
Scheperjans et al. [60]	Cross-sectional	*n =* 72 *PD* *n =* 72 *HC*	Fecal samples	16S rRNA miseq sequencing	On average, the abundance of *Prevotellaceae* in the feces of PD patients was reduced by 77.6% as compared to the controls. Relative abundance of *Prevotellaceae* of 6.5% or less had 86.1% sensitivity and 38.9% specificity for PD.
Cerroni et al. [61]	Longitudinal	*N =* 18 *PD* *N =* 18 *HC*	Fecal samples	16S rRNA amplicons analysis	No differences in the gut microbiota (beta diversity) structure at the follow-up in both PD patients and HC were observed, which remained stable for both patients and the controls. These results suggest that the gut microbiota may remain stable over a period of 14 months.
Unger et al. [62]	Longitudinal	*n =* 34 *PD* *n =* 34 *HC*	Fecal samples	Quantitative PCR	Fecal SCFA concentrations were significantly reduced in PD patients as compared to the controls. The bacterial phylum *Bacteroidetes* and the bacterial family *Prevotellaceae* were reduced, *Enterobacteriaceae* were more abundant in fecal samples from PD patients as compared to matched controls.

### 3.4. Quality Appraisal

The overall risk of bias evaluation for included papers is summarized in Table 3. Nine studies showed an unclear risk of bias. One study included did not allocate for all patients included at the start of the research in their results [48]. In other studies (75%), the treatment of patients throughout the duration was not described. We deemed the evidence related to the primary outcomes of moderate quality, as next-generation sequencing was utilized and established analyses were integrated to generate the data.

## 4. Discussion

This systematic review and meta-analysis aimed to further investigate the relationship between the microbiome and cognition and discuss the implications of this in the context of aging. The findings corroborate previous evidence and substantiate the hypothesis that significant alterations are observed in individuals with cognitive impairments, including those with Parkinson’s disease, dementia, and Alzheimer’s disease. The underlying facilitator for the relationship between cognition and the gut microbiome is the microbiome-gut-brain axis [52]. One study conducted by Ma et al. at Harvard University examined the relationship between the gut microbiome, bowel movement frequency, and subjective cognition in 515 women and men. The study found that individuals with specific microbial profiles in the gut, including more bacteria that can cause inflammation and fewer bacteria responsible for digesting dietary fibers, had less frequent bowel movements and worse cognitive function [63]. Another study conducted by Komanduri et al. explored the relationship between the gut microbiome and cognition in older Australians. The study identified specific families of bacteria that were associated with different domains of cognition, such as episodic secondary memory, working memory, and concentration [64].

Chronic constipation has also been associated with cognitive decline. A study published in the New Scientist found a correlation between chronic constipation and cognitive decline. However, the study did not test the causal relationship between bowel movements, the gut microbiome, and cognition [47,65,66].

Probiotics, which are beneficial bacteria, seem to have a positive effect in improving cognitive function and preventing cognitive decline associated with aging. A study published in Neuroscience News found that participants with mild cognitive impairment who received the probiotic *Lactobacillus rhamnosus GG* for three months showed improved cognitive scores. The study also observed changes in the gut microbiome, with a decrease in the abundance of *Prevotella bacteria*, which was associated with cognitive improvement. Certain molecules produced by gut bacteria may modulate the functionality of neuroprotective hormones that can cross the blood-brain barrier [67,68].

A systematic review by Cooke et al. examined the influence of the microbiome on cognition and stress. The findings mirrored that of our systematic review, highlighting the correlations between the diversity of the microbiome composition and areas of the brain related to cognitive functions, specifically memory and visual processing. Similarly, intervention studies targeting the gut microbiota, evaluating the resulting cognition of individuals, resulted in improved brain activity, cognition, depression, and anxiety. Collectively, this warranted the conclusion that the gut microbiome may have a substantial role in brain connectivity and cognitive performance, thus modulating this diverse community poses a promising approach to enhancing brain connectivity and emotional well-being and reducing cognitive decline [69].

In addition to the impact of the microbiome on cognition, this systematic review and meta-analysis also observed evidence that suggests a relationship exists between the composition of the gut microbiota and the presentation of symptoms in neurodegenerative disorders, such as Parkinson’s disease, Alzheimer’s disease, and dementia.

### 4.1. Impact of the Microbiome on Parkinson’s Disease

Parkinson’s disease may begin in the gut: Recent research published in the journal Cell suggests that Parkinson’s disease may begin in the gut, not the brain. The study found that the disease is triggered by bacteria that live in the human gastrointestinal tract. This opens up a new avenue of inquiry into the disease [70].

A systematic review of case-control studies found that there were consistent alterations in the gut microbiome of Parkinson’s patients compared to the non-Parkinson’s controls. These alterations included increased abundances of certain bacterial genera and reduced abundances of others. Additionally, some bacterial genera were found to correlate with Parkinson’s motor severity, motor response complications, and cognitive function [71].

Scheperjans et al. observed a positive relationship between the abundance of *Enterobacteriaceae* and the severity of postural instability and gait difficulty in individuals with Parkinson’s disease. Collectively, this suggests that the intestinal microbiome is altered in this neurological condition and is related to motor phenotype [60,72]. Minato et al. also reported that in individuals with deteriorated Parkinson’s disease, as indicated by the Unified Parkinson’s Disease Rating Scale (UPDRS), lower Bifidobacterium counts (*q* < 0.05) could be detected as compared to the stable group. This may suggest that lower counts of specific microbiota, such as *Bifidobacterium*, may be predictive of disease progression in two years [52].

A similar meta-analysis to that presented here was performed by Hirayama and Ohno, who exclusively investigated the relationship between Parkinson’s disease and the microbiome across 20 studies. The findings indicated two pathomechanisms in the intestine of individuals with Parkinson’s disease. A notable decrease in SCFA-producing bacteria and an increase in mucin-degrading bacteria were described, leading to the hypothesis of increased intestinal permeability. Moreover, the decrease in SCFA-producing bacteria may aggravate microglia-mediated inflammation in the CNS [73]. Similar results were presented by Unger et al. in this review [62]. Therefore, in individuals with Parkinson’s disease, the gut microbiome, specifically SCFA-producing bacteria, and mucin-degrading bacteria, may represent a novel therapeutic target.

The identification of specific bacterial changes in the gut microbiome of Parkinson’s patients may have implications for the treatment of the disease. Targeting these specific changes through interventions such as probiotics or fecal microbiota transplantation (FMT) could potentially be explored as therapeutic approaches [74].

### 4.2. Influence of the Microbiome on Alzheimer’s Disease and Dementia

Several research studies have indicated that changes in the human microbiome, particularly the gut microbiome, may precede cognitive declines associated with Alzheimer’s disease. In one study, participants who showed early signs of Alzheimer’s showed a significantly different composition of gut bacteria as compared to healthy controls. These changes in the microbiome were correlated with beta-amyloid and tau levels in the brain, which are key markers of Alzheimer’s disease. However, the study did not find a relationship between these microbiome changes and degenerative changes in the brain, which generally occur later in the progression of Alzheimer’s. The same study also suggested that differences in the gut microbiome could be a cause, or an outcome, of the brain changes observed in Alzheimer’s disease. This opens up the possibility that treatments such as probiotics or fecal transplants, which promote the growth of beneficial bacteria in the gut, could slow the development of Alzheimer’s and mitigate its more severe symptoms [75,76].

This opens the possibility that treatments with probiotics or fecal transplants, promoting the growth of beneficial bacteria in the gut, may slow the development of Alzheimer’s and alleviate its most severe symptoms. Despite these findings, a secondary goal of ongoing research is to determine whether there is a correlation between microbiome identity/abundance and measures of cognition in patients with normal control, mild cognitive impairment, and Alzheimer’s disease [77].

The gut microbiome appears to play a significant role in Alzheimer’s disease and dementia, but further research is needed to fully understand this relationship and develop effective treatments based on these findings.

In individuals with advanced dementia, it is evident that the diversity of the microbiome is substantially reduced as compared to healthy controls [48]. It was observed that, at the phylum level, there were mild insignificant decreases in *Firmicutes* and *Actinobacteria*, whilst small but insignificant increases were noted in *Bacteroidetes* [41,78]. However, an opposite trend was described by Jemimah et al. in a similar meta-analysis, reporting an insignificant but moderate increase in the *Firmicutes* phylum and a moderate decrease in the *Bacteroidetes* phylum in individuals with Alzheimer’s disease or mild cognitive impairment [79]. Nevertheless, both Vogt et al. and Jemimah et al. described higher amounts of the genus *Bilophila* of the phylum *Proteobacteria* in individuals with Alzheimer’s disease [36,41,79]. Furthermore, at the genus level, *Ruminococcus*, *Bilophila*, *Desulfovibrio*, *Barnesiella*, *Butyricimonas*, *Acidaminococcus*, *Pyramidobacter*, and *Oxalobacter* were negatively associated with measures of cognition function [58].

### 4.3. Additional Considerations and Future Research

Several health-related factors were described in the identified literature that may exacerbate the influence of the gut microbiome on cognitive function: for example, antropometric parameters (body mass index, BMI). Different studies have suggested that obesity, often indicated by a high BMI, is associated with a different composition and diversity of the gut microbiota, as compared to lean individuals [80,81]. Obesity has been linked to a higher ratio of *Firmicutes* to *Bacteroidetes* in the gut microbiome [82]. This altered composition of gut bacteria may contribute to the development of obesity by affecting energy extraction from the diet and fat accumulation in the body [83]. In contrast, a diverse microbiome, often found in lean individuals, is associated with a lower ratio of *Firmicutes* to *Bacteroidetes* [81]. In addition, changes in the gut microbiome related to obesity can affect the brain through the microbiota-gut-brain axis, a bidirectional communication pathway [84]. The gut microbiota can produce metabolites, such as short-chain fatty acids (SCFAs) and neurotransmitters that can cross the blood-brain barrier, thus affecting brain function [85]. Dysbiosis, or an imbalance of the gut microbiota, associated with obesity could lead to the altered production of these metabolites, influencing cognitive function [86]. Moreover, the gut microbiome can affect the immune system, and an altered gut microbiome in obesity can lead to chronic low-grade inflammation, which is known to negatively impact cognitive functions [87].

Cattaneo et al. observed a significant association between the abundance of *P. aeruginosa* and BMI among individuals with brain amyloidosis. Similarly, other factors exert an effect on cognitive function, such as the pro-inflammatory cytokines NLRP3, CXCL2, IL-6, and IL-1B, considered inflammatory biomarkers [51]. The role of inflammatory pathways in microbiome dysbiosis and neurodegenerative disorders was also discussed by Haran et al. In their analysis, associations between the dysregulation of the anti-inflammatory P-glycoprotein pathway, the microbiome, and the pathogenesis of Alzheimer’s disease were observed [56,88]. The diet also plays a significant role in the microbiome composition, as supported by Saji et al., who observed that adherence to a traditional Japanese diet was inversely associated with cognitive decline and was, in general, associated with low concentrations of different gut microbial metabolites [54]. Further studies are needed to determine the underlining relationships between cognitive decline and microbiome.

## 5. Conclusions

In summary, this systematic review and meta-analysis were undertaken to probe the intricate relationship between the microbiome and cognition, with a particular focus on its implications in the aging process. The outcomes not only harmonize with prior evidence but also bolster the notion that substantial changes emerge in individuals facing cognitive impairments, encompassing conditions such as Parkinson’s disease, dementia, and Alzheimer’s disease. Central to the amalgamation of cognition and the gut microbiome is the microbiome-gut-brain axis, a nexus of interaction that underscores the significance of our findings. However, it is important to acknowledge certain limitations and avenues for future investigation. Notably, the diminished diversity of the microbiome observed in advanced dementia cases stands in contrast to healthy controls. Furthermore, the interplay of diverse factors, such as body mass index (BMI), obesity, and inflammation, intricately contributes to the role of the microbiome in cognitive function. Further exploration is imperative to gain a comprehensive grasp of these complex relationships.

In conclusion, this systematic review and meta-analysis illuminate the interwoven relationship between the microbiome and cognition, particularly in the context of aging and neurodegenerative conditions. The outcomes underscore the critical role of the microbiome in cognitive well-being and its potential as a therapeutic avenue to enhance brain connectivity and ameliorate cognitive decline. While this review signifies a substantial advancement in our understanding, it is evident that further research is requisite to meticulously decipher the intricate mechanisms that underpin these interdependent relationships.

## Figures and Tables

**Table 1:** Phyla, Major Families, and Genera in the Microbiome of Age-related Cognitive Decline.

Phylum	Major Families	Major Genera
Firmicutes	Lachnospiraceae, Ruminococcaceae, Clostridiaceae	Faecalibacterium, Roseburia
	Erysipelotrichaceae	Eubacterium, Clostridium
Bacteroidetes	Bacteroidaceae, Prevotellaceae	Bacteroides, Prevotella
	Rikenellaceae, Porphyromonadaceae	Parabacteroides, Alistipes
Actinobacteria	Bifidobacteriaceae	Bifidobacterium
	Coriobacteriaceae	Collinsella
Proteobacteria	Enterobacteriaceae	Escherichia, Klebsiella
	Desulfovibrionaceae	Desulfovibrio

**Table 3:** GRADE criteria for risk of bias evaluation. Green: No Risk of Bias; Yellow: Maybe Risk of Bias; Red: High Risk of Bias.

Reference	Methodological Quality	Directness of Evidence	Heterogeneity	Precision of Effect Estimates	Publication Bias	Level of Evidence	Recommendation for Use
Cattaneo et al. [51]						Moderate	Moderate
Mitano et al. [52]						Moderate	Moderate
Vogt et al. [41]						Moderate	Moderate
Araos et al. [48]						Low	Low
Nguyen et al. [53]						Moderate	Moderate
Saji et al. [54]						Moderate	Low
Qian et al. [55]						Moderate	Moderate
Zhuang et al. [50]						Moderate	Moderate
Haran et al. [56]						Moderate	Low
Liu et al. [49]						Moderate	Moderate
Saji et al. [57]						Moderate	Low
Ren et al. [58]						Moderate	Moderate
Liu et al. [59]						Moderate	Moderate
Scheperjans et al. [60]						Moderate	Moderate
Cerroni et al. [61]						Moderate	Moderate
Unger et al. [62]						Moderate	Moderate

## Data Availability

Data is contained within the article or supplementary material.

## Supplementary Materials

The following supporting information can be downloaded at: https://www.mdpi.com/article/10.3390/ijms241813680/s1, reference [89] is cited in there.
