# Supplementary material for: Age-Related Cognitive Decline, Focus on Microbiome: A Systematic Review and Meta-Analysis
Source: Int J Mol Sci. 2023 Sep 5;24(18):13680. doi: 10.3390/ijms241813680 (PMC10531012; doi:10.3390/ijms241813680)
Supplement: Supplementary file 1 [file ijms-24-13680-s001.zip › ijms-2562350-supplementary.pdf]

PRISMA 2020 Checklist

Section and Topic	Item #	Checklist item	Location where item is reported
**TITLE**			
Title	1	Identify the report as a systematic review.	Page 1
**ABSTRACT**			
Abstract	2	See the PRISMA 2020 for Abstracts checklist.	Page 1
**INTRODUCTION**			
Rationale	3	Describe the rationale for the review in the context of existing knowledge.	Pages 1-4
Objectives	4	Provide an explicit statement of the objective(s) or question(s) the review addresses.	Page 3-4
**METHODS**			
Eligibility criteria	5	Specify the inclusion and exclusion criteria for the review and how studies were grouped for the syntheses.	Page 4
Information sources	6	Specify all databases, registers, websites, organisations, reference lists and other sources searched or consulted to identify studies. Specifythe date when each source was last searched or consulted.	Page 4
Search strategy	7	Present the full search strategies for all databases, registers and websites, including any filters and limits used.	Page 4
Selection process	8	Specify the methods used to decide whether a study met the inclusion criteria of the review, including how many reviewers screened eachrecord and each report retrieved, whether they worked independently, and if applicable, details of automation tools used in the process.	Page 4
Data collection process	9	Specify the methods used to collect data from reports, including how many reviewers collected data from each report, whether they worked independently, any processes for obtaining or confirming data from study investigators, and details of automation tools used in the process.	Page 4
Data items	10a	List and define all outcomes for which data were sought. Specify whether all results that were compatible with each outcome domain in eachstudy were sought (e.g. for all measures, time points, analyses), and if not, the methods used to decide which results to collect.	Page 4-5

	10b	List and define all other variables for which data were sought (e.g. participant and intervention characteristics, funding sources). Describe anyassumptions made about any missing or unclear information.	Page 4-5
Study risk of bias assessment	11	Specify the methods used to assess risk of bias in the included studies, including details of the tool(s) used, how many reviewers assessedeach study and whether they worked independently, and if applicable, details of automation tools used in the process.	Page 5
Effect measures	12	Specify for each outcome the effect measure(s) (e.g. risk ratio, mean difference) used in the synthesis or presentation of results.	Page 4
Synthesis methods	13a	Describe the processes used to decide which studies were eligible for each synthesis (e.g. tabulating the study intervention characteristicsand comparing against the planned groups for each synthesis (item #5)).	Page 4
	13b	Describe any methods required to prepare the data for presentation or synthesis, such as data conversions.	Page 4
Reporting bias assessment	14	Describe any methods used to assess risk of bias due to missing results in a synthesis (arising from reporting biases).	Pages 5
Certainty assessment	15	Describe any methods used to assess certainty (or confidence) in the body of evidence for an outcome.	Page 5
**RESULTS**			

PRISMA 2020 Checklist

Section and Topic	Item #	Checklist item	Location where item is reported
Study selection	16a	Describe the results of the search and selection process, from the number of records identified in the search to the number of studies included in the review, ideally using a flow diagram.	Figure 1
	16b	Cite studies that might appear to meet the inclusion criteria, but which were excluded, and explain why they were excluded.	Figure 1
Results of syntheses	20a	For each synthesis, briefly summarise the characteristics and risk of bias among contributing studies.	Pages 5-8
	20b	Present results of all statistical syntheses conducted. If meta-analysis was done, present for each the summary estimate and its precision (e.g. confidence/credible interval) and measures of statistical heterogeneity. If comparing groups, describe the direction of the effect.	Figures 2-5
	20c	Present results of all investigations of possible causes of heterogeneity among study results.	Pages 4-8
	20d	Present results of all sensitivity analyses conducted to assess the robustness of the synthesized results.	Pages 4-8
Reporting biases	21	Present assessments of risk of bias due to missing results (arising from reporting biases) for each synthesis assessed.	Pages 4-8
Certainty of evidence	22	Present assessments of certainty (or confidence) in the body of evidence for each outcome assessed.	Pages 4-8
**DISCUSSION**			
Discussion	23a	Provide a general interpretation of the results in the context of other evidence.	Pages 13-15
	23b	Discuss any limitations of the evidence included in the review.	Pages 13-15
	23c	Discuss any limitations of the review processes used.	Pages 13-15
	23d	Discuss implications of the results for practice, policy, and future research.	Pages 13-15
**OTHER INFORMATION**			
Support	25	Describe sources of financial or non-financial support for the review, and the role of the	Page 16

		funders or sponsors in the review.	
Competing interests	26	Declare any competing interests of review authors.	Page 16
Availability of data	27	Report which of the following are publicly available and where they can be found: template data collection forms; data extracted from includedstudies; data used for all analyses; analytic code; any other materials used in the review.	NA

From: Page MJ, McKenzie JE, Bossuyt PM, Boutron I, Hoffmann TC, Mulrow CD, et al. The PRISMA 2020 statement: an updated guideline for reporting systematic reviews. BMJ 2021;372:n71. doi: 10.1136/bmj.n71

For more information, visit: http://www.prisma-statement.org/

Search strategy for all databases

1. Microbiome ratio
2. Gut Microbiome ratio
3. Cognition ratio
4. Cognitive Decline ratio
5. Alzheimer's Disease
6. Parkinson's Disease
7. Dementia
8. Neurodegenerative Disorder
7. 1 AND 4
8. 1 AND 5
9. 1 AND 6
10. 1 AND 7
11. 1 AND 8
12. 2 AND 4
13. 2 AND 5
14. 2 AND 6
15. 2 AND 7
16. 2 AND 8
17. 3 AND4
18. 3 AND 5
19. 3 AND 6
20. 3 AND 7
21. 3 AND 8
22. 4 AND 5
23. 4 AND 6
24. 4 AND 7
25. 4 AND8
26. 1 OR 2 OR 3 OR 4
27. 5 AND 25
28. 6 AND 25
29. 7 AND 25
30. 8 AND 25
31. 5 OR 6 OR 7 OR 8
32. 1 AND 30
33. 2 AND 30
34. 3 AND 30
35. 4 AND 30
36. 25 AND 30

PRISMA 2020 for Abstracts Checklist

Section and Topic	Item #	Checklist item	Reported (Yes/No)
**TITLE**			
Title	1	Identify the report as a systematic review.	Yes
**BACKGROUND**			
Objectives	2	Provide an explicit statement of the main objective(s) or question(s) the review addresses.	Yes
**METHODS**			
Eligibility criteria	3	Specify the inclusion and exclusion criteria for the review.	No (space constraints)
Information sources	4	Specify the information sources (e.g. databases, registers) used to identify studies and the date when eachwas last searched.	Yes
Risk of bias	5	Specify the methods used to assess risk of bias in the included studies.	Yes
Synthesis of results	6	Specify the methods used to present and synthesise results.	No (space constraints)
**RESULTS**			
Included studies	7	Give the total number of included studies and participants and summarise relevant characteristics ofstudies.	Yes
Synthesis of results	8	Present results for main outcomes, preferably indicating the number of included studies and participants foreach. If meta-analysis was done, report the summary estimate and confidence/credible interval. If comparing groups, indicate the direction of the effect (i.e. which group is favoured).	Yes
**DISCUSSION**			
Limitations of evidence	9	Provide a brief summary of the limitations of the evidence included in the review (e.g. study risk of bias,inconsistency and imprecision).	Yes
Interpretation	10	Provide a general interpretation of the results and important implications.	Yes
**OTHER**			
Funding	11	Specify the primary source of funding for the review.	NA
Registration	12	Provide the register name and registration number.	Yes

From: Page MJ, McKenzie JE, Bossuyt PM, Boutron I, Hoffmann TC, Mulrow CD, et al. The PRISMA 2020 statement: an updated guideline for reporting systematicreviews. BMJ 2021;372:n71. doi: 10.1136/bmj.n71

For more information, visit: http://www.prisma-statement.org/

Synthesis Without Meta-analysis (SWiM) Table:

Study Characteristics	Results and Findings
**Population**	A total of 1303 participants were included in the studies. This encompassed individuals with various cognitive conditions, including Alzheimer's disease, dementia, Parkinson's disease, brain amyloidosis, post-stroke cognitive impairments, and healthy/matched controls.
**Biospecimen**	The biospecimen collected across all studies was fecal samples.
**Microbiological Analysis**	Common methods for analyzing the gut microbiota composition included 16S rRNA sequencing (56% of studies), T-RFLP analysis (13% of studies), and qRT-PCR (13% of studies).
**Relative Abundance**	Significant alterations in the relative abundance of certain phyla, including Firmicutes, Bacteroidetes, Actinobacteria, and Proteobacteria, were observed in individuals with cognitive impairments compared to healthy controls.
**Dominant Phyla in Alzheimer's/Dementia**	Individuals with Alzheimer's disease or dementia exhibited higher dominance of Bacteroidetes and Firmicutes phyla.
**Heterogeneity**	Meta-analyses of relative abundance in Alzheimer's disease showed substantial heterogeneity, underscoring the complex nature of the gut microbiome-cognition relationship.

Quality Appraisal: Overall, the risk of bias assessment revealed that nine studies had an unclear risk of bias, with certain studies lacking patient allocation and detailed treatment descriptions. The evidence quality related to primary outcomes was rated as moderate, supported by the use of advanced sequencing methods and established analyses.
